# The prevention of delirium in elderly with obstructive sleep apnea (PODESA) study: protocol for a multi-centre prospective randomized, controlled trial

**DOI:** 10.1186/s12871-017-0465-5

**Published:** 2018-01-03

**Authors:** Jean Wong, David Lam, Stephen Choi, Mandeep Singh, Naveed Siddiqui, Sanjeev Sockalingam, Frances Chung

**Affiliations:** 1 Department of Anesthesia, Toronto Western Hospital, University Health Network, University of Toronto, 399 Bathurst Street, Toronto, ON M5T 2S8 Canada; 2 Department of Anaesthesia, Sunnybrook Health Sciences Center, University of Toronto, Toronto, ON Canada; 3 Toronto Sleep and Pulmonary Center, Toronto, ON Canada; 4 Department of Anaesthesia, Mount Sinai Hospital, University of Toronto, Toronto, ON Canada; 5 Centre for Mental Health, University Health Network, Department of Psychiatry, University of Toronto, Toronto, ON Canada

**Keywords:** Aged, Continuous positive airway pressure, Delirium, Sleep apnea, Obstructive, Perioperative period

## Abstract

**Background:**

Delirium is a common problem that occurs in 5–50% of elderly individuals following surgery. Patients who develop delirium after surgery are at increased risk for serious complications. Recent studies suggest that patients with obstructive sleep apnea (OSA), a sleep disorder characterized by repeated episodes of complete or partial blockage of the upper airway – are at greater risk to develop delirium. OSA is more common in elderly individuals but is often undiagnosed. Identification and treatment of unrecognized OSA may reduce the incidence of postoperative delirium. However, few studies have investigated the effect of perioperative treatment of OSA to prevent postoperative delirium.

**Methods:**

This multi-centre randomized controlled trial will enrol 634 elderly patients undergoing elective hip/knee replacement surgery. The study has been approved by the Research Ethics Boards of the three participating institutions. Patients will be screened with the STOP-Bang questionnaire. Those with a score of 3 or greater will have a portable home sleep study using the ApneaLink™ Air device. Patients identified to have OSA will be randomized to 1) Auto-titrating continuous positive airway pressure (APAP) applied during sleep for 72 h after surgery or until discharge if they are discharged before 72 h **or** 2) Control group – routine care, no APAP. All patients will be evaluated for delirium for 72 h after surgery or until discharge if they are discharged before 72 h. The primary outcome is the occurrence of delirium – assessed twice daily using the Confusion Assessment Method for 72 h or until discharge if the hospital stay is <72 h.

**Discussion:**

Delirium is associated with increased morbidity and mortality, and higher healthcare costs. With the aging population, the incidence of postoperative delirium will likely increase as the number of elderly individuals undergoing surgery rises. The results of our study will be published in a peer-reviewed journal and presented at local and international medical conferences. Our study findings may lead to improved surgical outcomes, enhanced patient safety and reduced healthcare costs.

**Trial registration:**

This study was retrospectively registered at clinicaltrials.gov NCT02954224 on November 3, 2016.

## Background

### Postoperative delirium

Delirium is an acute confusional state, with fluctuating impairment in attention and decreased awareness of one’s environment [1, 2]. Postoperative delirium is a common complication after major surgery; the incidence has been reported to be 13–41% after elective hip or knee replacements, and 26–61% for patients undergoing femoral neck fractures [3, 4]. Postoperative delirium is associated with increased mortality, morbidity, longer hospital stays [5], higher hospital costs [6], cognitive deterioration, and delayed rehabilitation. The pathophysiology of delirium remains poorly understood. The development of postoperative delirium is thought to be a multi-factorial process [7] with a combination of predisposing and precipitating factors. Predisposing factors can be defined as the patient’s baseline vulnerabilities and precipitating factors as injury that occurs throughout the perioperative period [8]. Predisposing factors include sex, age, cognitive status, and coexisting medical conditions, while precipitating factors include surgery, and hypoxia, however, there are few effective preventive measures for delirium [9]. The clinical presentation of delirium varies [10] but postoperative delirium typically occurs on days 2 and 3 after surgery [11, 12].

### Obstructive sleep apnea (OSA)

OSA is a sleep disorder characterized by repeated episodes of complete or partial blockage of the upper airway, resulting in cessation of airflow for at least 10 S*. OSA* is a serious medical condition that is associated with hypertension, stroke, myocardial infarction, diabetes, long term cognitive impairment and lower quality of life [13, 14]. OSA is more prevalent in elderly individuals [15]. In fact, 17% of men and 9% of women aged 50–70 years old are estimated to have OSA [16]. OSA may be under-reported in elderly individuals because some of the symptoms for OSA such as tiredness may be attributed to normal aging.

### Clinical studies of OSA and postoperative delirium

An association between OSA and postoperative delirium was reported in a study on elderly patients undergoing elective knee replacement surgery [11]. In a study of patients undergoing elective cardiac surgery, a preoperative diagnosis of moderate to severe OSA was associated with a 6-fold increased risk of postoperative delirium [17]. In a retrospective study of matched control patients undergoing hip and knee replacement surgery, the incidence of delirium was 2-fold higher and the length of hospital stay was longer, in patients with OSA vs. those without OSA [18]. There are also several case reports of patients with acute delirium and OSA that resolved with treatment of OSA by continuous positive airway pressure (CPAP) [19–21]. A recent trial of elderly surgical patients at risk for OSA who were randomly assigned to either CPAP or routine care reported that OSA and apnea are associated with postoperative delirium, however, they did not find a reduction in postoperative delirium with CPAP [22]. It should be noted that in this trial, the diagnosis of OSA was not confirmed with a sleep study and the compliance with CPAP was low – which may have led to the lack of a difference between the CPAP and no CPAP group. Therefore, it is unclear from previous studies whether diagnosis and treatment of OSA with CPAP will decrease the incidence of post-operative delirium in elderly patients. Precipitating factors for postoperative delirium in patients with untreated OSA may include hypoxia and disrupted sleep that is worsened by inflammation from surgery, pain and opioid analgesics.

### Treatment of OSA and auto-titrating CPAP

Among the different treatments for OSA, auto-titrating continuous positive airway pressure (APAP) is widely used. It is highly effective in eliminating OSA, and alleviating its symptoms including daytime sleepiness, cognitive function and overall quality of life [23, 24]. APAP adjusts the pressure delivered to the patient based on a breath-by-breath measurement of airflow to maintain the minimal pressure required to keep the upper airway unobstructed. Studies have reported that starting treatment with APAP can be just as effective as CPAP titrated during polysomnography (PSG) [25–27].

### Objectives

The objectives of this trial are to identify OSA preoperatively with a portable home sleep study and to determine whether APAP treatment of OSA will decrease the incidence of post-operative delirium in elderly individuals undergoing elective hip or knee replacement surgery.

## Methods

### Trial design

**Fig. 1:**
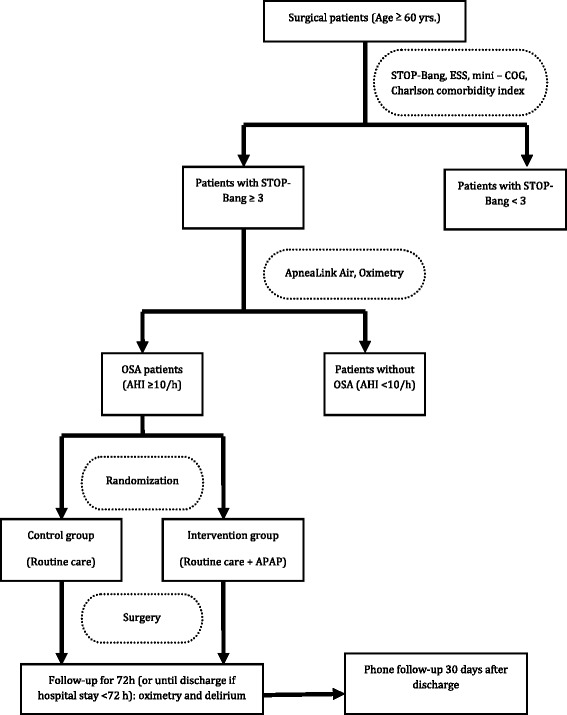
Flow chart of study. ESS: Epworth Sleepiness Scale; OSA: obstructive sleep apnea; APAP: auto-titrating continuous positive airway pressure; AHI: apnea-hypopnea index

The **P**revention **O**f **D**elirium in **E**lderly with Obstructive **S**leep **A**pnea (PODESA) study is a multi-centre, prospective, randomized controlled superiority trial with two parallel groups. Randomization at each site will be performed with a 1:1 allocation into intervention (APAP) or control (routine care) group. The overall study design is shown in Fig. 1.

### Study setting

This ongoing study is being conducted at three academic hospitals: Toronto Western Hospital, University Health Network; Mount Sinai Hospital; and Sunnybrook Hospital located in Toronto, Ontario Canada over 3 years. The target completion date is December 2018.

### Eligibility criteria

Inclusion criteria for participation include patients who are: 1) 60 years and older; 2) scheduled for elective hip or knee replacement surgery at least 4 working days after the preoperative clinic visit; 3) possess the cognitive and physical capability necessary to comprehend and complete the study questionnaires; 4) proficient in English with a reading level at Grade 6 (patient or accompanying person); 5) accessible for follow-up via telephone, or via the Internet; and 6) able to provide informed consent.

The exclusion criteria include patients with schizophrenia or active psychosis within the last 3 months, current use of antipsychotic medication, anxiety disorders, poorly controlled depression, multiple psychiatric disorders, a history of drug or alcohol dependence or abuse within the last 3 months, dementia, and/or clinically significant neurological disorders (stroke, epilepsy, brain tumours, Parkinson’s Disease etc.). Other exclusion criteria include patients undergoing surgery that is two-staged involving more than one surgical procedure to be performed within the same hospitalization period, central sleep apnea (more than 50% central events, or with significant Cheyne-Stokes respiration pattern), significant cardiac disease (New York Heart Association functional class III and IV, severe valvular heart disease, dilated cardiomyopathy, implanted cardiac pacemaker, unstable angina), myocardial infarction or cardiac surgery or percutaneous coronary intervention within 3 months, severe tracheal or lung disease, or a contraindication to the APAP facemask. Patients with a prior diagnosis of sleep-related breathing disorder who are compliant with CPAP/APAP treatment will be excluded. However, patients who have had prior sleep studies may be included if they have been lost to follow-up by a sleep physician, are not on treatment or are non-compliant to treatment (mean CPAP or APAP nightly use <4 h, or median nightly CPAP or APAP use <50% of total sleep time) [28].

### Patient screening and baseline assessment

Trained research assistants will screen patients who are scheduled for elective hip or knee replacement surgery in each site for eligibility to participate in the study. There will be no financial or non-financial incentives provided to participants for enrolment. Those interested in participating in the study will be given written information about the study, including the objectives and procedures. Research assistants will ask patients to provide written informed consent prior to collection of the baseline assessment.

In the preoperative clinics, all patients who consent will complete the STOP-Bang questionnaire [29, 30], Epworth Sleepiness Scale [31], Charlson comorbidity index [32], and Mini-Cog Test [33, 34]. The STOP- Bang Questionnaire is a validated questionnaire to screen patients for OSA [29]. If a patient has a STOP-Bang score of 3 or higher, they will have a portable home sleep study with ApneaLink™ Air (ResMed, San Diego, California, USA) for one night. ApneaLink™Air is a Type III sleep apnea monitoring device that records the following data: nasal airflow, snoring, blood oxygen saturation, pulse and respiratory effort during sleep.

The sleep data will be manually scored and checked by a qualified sleep technologist, and interpreted by the study sleep physician for recordings of at least 3 h duration. In the case of inadequate sleep or any technical failure, the patient will repeat the Apnea LinkAir™ study once. The conduct, data retrieval and interpretation of the sleep study will be guided by the current Canadian Sleep Society [35] and American Academy of Sleep Medicine [36] guidelines for portable monitors. The scoring criteria for breathing events will be guided by the American Academy of Sleep Medicine definition [37]. An apnea is defined as a decrease in airflow by ≥90% of baseline for ≥10 s. A hypopnoea is defined as a decrease in airflow by >30% baseline for ≥10 s in association with either ≥3% oxygen desaturation from pre-event baseline and/or the event is associated with an arousal [37].

Oximetry monitoring with a high-resolution pulse oximeter wristwatch (PULSOX-300i, Konica Minolta Sensing, Inc., Osaka, Japan) will be carried out for one night before surgery and for three nights postoperatively or until discharge if the hospital stay is <72 h.

### Definition of OSA

Patients will be defined as having OSA if the apnea-hypopnea index (AHI) is ≥ 10/h on the ApneaLink™ Air study. Patients with AHI < 10/h will be excluded from further study.

### Interventions

Patients identified to have OSA (AHI ≥ 10/h) will be randomised to receive APAP or control (routine care).

APAP: APAP will be applied during day/night sleep for 72 h after surgery or until discharge if the hospital stay is <72 h. The ResMed AirSense 10 Autoset device (ResMed San Diego, California, USA) is the APAP device that will be used for the study. The Airview™ server (ResMed, San Diego, California, USA) will be used to monitor the APAP compliance, AHI, mask leakage and breathing events.

Control (routine care): will receive standard care by the health care team, and no APAP. The standard of care may include oxygen therapy, or any other procedures or consultations as deemed necessary by the health care team as needed.

### Assignment of interventions

A computer generated randomization list for each participating site will be created by a research analyst who is not involved in the study. The patient assignments will be placed in serially numbered, opaque-sealed envelopes corresponding with the randomization schedule for each site. The research assistant will open the envelope after the sleep study confirms the diagnosis of OSA for the patient.

### Blinding

It is not possible to provide an ‘active placebo’ for the control group since it is not possible to program the APAP to zero cm H_2_O. Statisticians and study investigators performing data analysis will remain blinded. If any adverse event or complication occurs necessitating discontinuing the study intervention, this will result in voluntary un-blinding of outcome assessors.

### Perioperative management of patients in both groups

There will be no change to the usual surgical, anaesthetic or postoperative pain management of patients in both groups at the participating centers.

There will be a phone follow up 30 days after discharge from hospital to document any complications.

### Primary outcomes

The primary outcome is postoperative delirium. The diagnosis of postoperative delirium will be based on using the Confusion Assessment Method (CAM) [2]. CAM has been validated against the Diagnostic and Statistical Manual Disorders Fourth Edition. All patients will be evaluated for delirium with the CAM 2 times daily, for 72 h or until discharge if the hospital stay is <72 h, by trained research assistants. These assessments will be done on post-operative nights 1, 2 and 3 or until discharge (if the hospital stay is <72 h), with at least 6 h between assessments. The first assessment for delirium will be performed when patients can be sufficiently aroused (Richmond Agitation and Sedation Score > −4) [38] in order to be assessed for delirium on the day of surgery [39, 40]. If the patient’s delirium persists beyond 72 h after surgery, he/she will be assessed with the CAM twice daily until the delirium resolves.

### Secondary outcomes

The secondary outcome measures include: length of stay, time to ambulate, perioperative complications {i.e. intensive care unit admission, hypoxia (SpO_2_ < 90%), re-intubation, myocardial infarction/ischemia, wound infection, deep vein thrombosis, pulmonary embolus, pneumonia, urinary tract infection, sepsis, stroke, etc.} occurring within 30 days after the surgical procedure.

### Standardization of training

All study research assistants who perform the assessments for delirium will be trained by a staff psychiatrist at University Health Network to administer the CAM. The research assistants will also attend a delirium training session for CAM offered at University Health Network. Once the trainees have met the requirements of their training, they will each conduct their first interview in the presence of a previously trained team member on a patient enrolled in the study.

### Adherence

Patients in the intervention group will be reminded in each study visit the importance of adherence to APAP therapy.

### Retention and withdrawal

Each study site will make every effort to maintain interest by supporting their participants throughout the perioperative period. Participants may withdraw from the study for any reason at any time.

### Sample size

Based on an expected incidence of postoperative delirium of 24% in untreated OSA patients and 9.9% in treated OSA patients [11, 18] assuming a power of 0.8 and alpha error of 0.05, 110 patients/group (220 in total) are required. If the dropout rate is 20%, 264 patients need to be randomized. In our previous study, 45.9% of patients who had PSG met AHI criteria for OSA [41]; therefore, to obtain 220 patients with OSA, we need 576 patients to successfully complete the sleep study. If we assume that the rate of withdrawal and testing failure is 10%, we need to recruit a total of 634 patients for ApneaLink™ Air testing.

### Data and statistical analysis

The primary outcome - the incidence of postoperative delirium in the APAP and control group will be compared and the significance of the difference will be assessed with Chi-square test. The secondary outcomes that will be compared between the two groups include: length of stay, time to ambulate, intraoperative and postoperative complications (i.e. respiratory, cardiovascular, death, etc.). An intention-to-treat and a per-protocol analysis will be used to compare the intervention (APAP) vs. control (routine care) group. Differences in the primary and secondary outcomes will be assessed using parametric and non-parametric tests where appropriate, and a *P*-value <0.05 will be considered statistically significant. SAS 9.3 will be used to perform the statistical analyses.

### Data monitoring

The principal investigator will be informed of serious adverse events as soon as they occur and will notify the data safety monitoring board (DSMB) within 24 h of notification. The site investigators at the participating sites will report serious adverse events, or unanticipated problems involving risks to participants to their research ethics board (REB) and to the principal investigator of the study at University Health Network. If these problems are thought to be associated with participating in the trial, then they will also be reported to the REBs at the other participating sites and to the chairperson of the DSMB.

A charter will guide the functions of the DSMB. The DSMB will provide independent oversight of the PODESA study and will review the study data for participant safety. The DSMB will include 3 independent experts who will examine accumulating data to ensure the integrity and safety of the study. The DSMB will make recommendations regarding the continuation, modification, or termination of the trial. As this is a low-risk study, there will be a provision for stopping the study for safety concerns, but not for efficacy or for futility. An interim analysis of adverse events in the study is planned after 50% of the study subjects are recruited and have completed follow-up. The study will be stopped for safety concerns if the interim analysis shows that the patients who are randomized to the APAP group have a higher incidence of perioperative complications including death, delirium, cardiovascular complications (e.g. myocardial infarction, congestive heart failure, stroke), respiratory complications (e.g. re-intubation, pneumonia, postoperative ventilation), etc. than the patients in the control group, *P* < 0.05.

DSMB members will have no direct involvement with the study investigators or intervention. Each DSMB member will sign a Conflict of Interest Statement to confirm that they do not have current affiliations, if any, with pharmaceutical and biotechnology companies (e.g., stockholder, consultant), and any other relationship that could be perceived as a conflict of interest related to the study and / or associated with commercial interests pertinent to study objectives.

The risks associated with this study are anticipated to be low. The potential risks to study participants include feeling uncomfortable with the use of the APAP mask. It is possible that the application of the APAP mask may cause skin irritation, dry mouth, nasal congestion, sneezing, or nasal drip. Participants will be informed of the risks during the informed consent process of the study and they will be able to report these risks during the preoperative adjustment period and 3 day postoperative follow up. Other potential risks include anxiety from dealing with a new diagnosis of OSA, and potential for breach of confidentiality.

The steering committee (JW, FC) developed the PODESA protocol and is responsible for data collection, management, final data set and publications. Modifications to the protocol that may impact the conduct of the study must be agreed on by the steering committee and prior to implementation, must be approved by the relevant REBs. Important protocol modifications will be communicated to relevant parties.

Care will be provided to participants suffering from harm related to participation in the study.

### Auditing

There is currently no routine auditing system planned. The study may be randomly selected for auditing by the Internal Quality Audit team at University Health Network. The clinical study site may be directed for auditing should a complaint or study related issue from participants, REB or government health regulators (Health Canada) arise.

### Confidentiality

Health information will only be shared with members of the research team. Original study forms and research charts will be kept on file at the participating study sites. All paper based documents and data will be stored in a locked cabinet in a locked research office. Electronic data will be stored in a password-protected electronic database that will be stored on the departmental network drive and only be accessible via password-protected departmental computers. A study code ID will be used to identify all participants, instead of names. The participants’ personal identifying information and names will be stored separately from the study data. Only the principal investigator, co-investigators, research assistants, and research statistician at the coordinating site will have access to the final trial dataset.

### Study organization

The PODESA study is coordinated by the Department of Anaesthesia at Toronto Western Hospital, University Health Network.

### Dissemination

The results of our study will be published in a peer-reviewed journal and presented at local hospitals, national and international medical conferences. There are currently no plans to grant public access to the full protocol, participant-level data set or statistical code. However, researchers who are interested in accessing the data set should contact the study steering committee.

## Discussion

According to the Canadian Institute for Health Information, there were 51,272 hip replacement surgeries, and 61,421 knee replacement surgeries performed in Canada for patients ≥ 65 years old in 2014–2015. Over the last five years, the increase in hip replacement and knee replacements has risen by 20.0% and 20.3% respectively. Over the next four decades, the population over age 60 is expected to double, and the incidence of postoperative delirium will likely increase exponentially as the number of elderly individuals requiring hospital admission for surgical reasons rises. Our study findings will increase knowledge about prevention and management of delirium in elderly patients undergoing surgery.

## Abbreviations

AHI: Apnea-hypopnea index
APAP: Auto-titrating continuous positive airway pressure
CAM: Confusion Assessment Method
CPAP: Continuous positive airway pressure
DSMB: Data safety monitoring board
OSA: Obstructive sleep apnea
PODESA: Prevention Of Delirium in Elderly with Obstructive Sleep Apnea
PSG: Polysomnography
REB: Research ethics board
